# ‘We’re getting there’: Registrar and examiner perspectives on structured oral examinations in emergency medicine

**DOI:** 10.4102/jcmsa.v3i1.206

**Published:** 2025-06-26

**Authors:** Sa’ad Lahri, Rhoda Meyer

**Affiliations:** 1 Division of Emergency Medicine, Faculty of Medicine and Health Sciences, Stellenbosch University, Cape Town, South Africa; 2 Department of Health Professions Education, Faculty of Medicine and Health Sciences, Stellenbosch University, Cape Town, South Africa

**Keywords:** online-structured oral examinations, emergency medicine, workplace-based assessments, web-based structured oral examinations, health professions education

## Abstract

**Background:**

Structured oral examinations (SOEs) are essential for assessing clinical competence in postgraduate emergency medicine qualifications. The rapid shift to web-based SOEs during the coronavirus disease 2019 pandemic and their continued use warrants an exploration of their effectiveness to identify areas for improvement in high-stakes assessments.

**Methods:**

A qualitative, exploratory approach situated within an interpretivist paradigm was used to explore the perspectives of registrars and examiners who had participated in a recent web-based Fellowship of the College of Emergency Medicine Part II examination in South Africa. Six registrars and seven examiners participated in semi-structured interviews. Reflexive thematic analysis, guided by Braun and Clarke’s approach, was used to identify key themes from the data.

**Results:**

Participants recognised the value of SOEs in assessment but also highlighted areas for improvement. Concerns included misalignment between exam content and clinical reasoning, anxiety because of the high-stakes nature of the assessment and familiarity with examiners and challenges faced by non-native English speakers. Examiners recommended targeted training in question design and bias mitigation.

**Conclusion:**

Enhancing SOEs through better examiner training and bias mitigation will strengthen their effectiveness. Integrating workplace-based assessment (WBAs) will reduce reliance on SOEs, promoting a more comprehensive approach to assessment in emergency medicine.

**Contribution:**

This study offers practical recommendations for improving examiner training, design and fairness in SOEs. Integrating WBAs with SOEs supports continuous, real-world assessment of competence. Bias-awareness training enhances equity, enabling institutions to design fairer, inclusive assessments.

## Introduction

Assessment is a cornerstone of health professions education (HPE), especially in high-stakes fields like emergency medicine, where clinical decisions can have immediate, life-altering consequences. As education scholar David Boud observed, students may be able to ‘escape from the effects of poor teaching’, but they ‘cannot (by definition if they want to graduate) escape the effects of poor assessment’.^1^ This insight underscores the critical role of rigorous, fair and meaningful assessment in shaping professional competence and protecting patient safety. Furthermore, assessment is integral to the learning process, where constructive alignment between learning outcomes, learning opportunities and assessment methods is essential for effective learning.^2^

Structured oral examinations (SOEs) have emerged as a vital tool for assessing the clinical aptitude and decision-making skills required in emergency medicine.^3^ Structured oral examinations provide a standardised approach to assessment by engaging registrars in structured discussions based on predefined scenarios.^4^ These examinations, which do not involve live patients, rely on carefully constructed checklists to guide the questioning process, ensuring that examiners systematically document and score student responses.^4^ The structured nature of SOEs is intended to minimise subjectivity and improve the fairness and consistency of assessments, which is crucial in ensuring that only competent practitioners are certified to practice in high-stakes environments like emergency medicine.^3,4^

The recent global pandemic necessitated a shift in how SOEs are administered, leading to the adoption of web-based formats, which continue to be used in South African postgraduate emergency medicine examinations.^5^ This adaptation, driven by the need to continue assessing key competencies remotely, involves presenting case scenarios through digital platforms to evaluate diagnostic reasoning, clinical decision-making and patient management skills.^5^ Following the pandemic, the Colleges of Medicine of South Africa (CMSA) opted to retain the web-based SOE format because of its demonstrated feasibility, examiner and candidate acceptability and logistical efficiency.^5^ This decision was applied across specialities, including emergency medicine, as part of a broader CMSA mandate to continue leveraging the benefits of virtual assessments.

**FIGURE 1:**
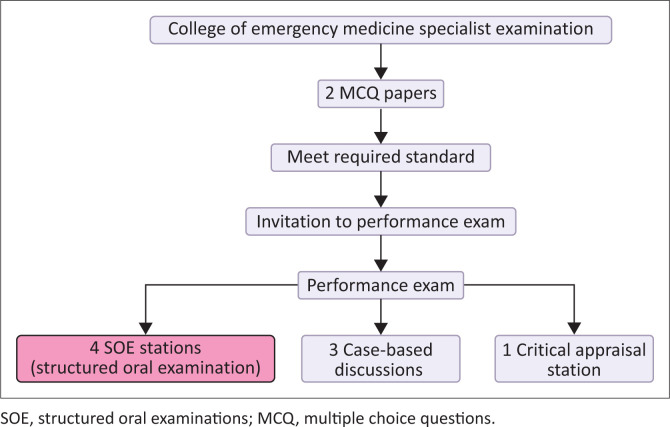
Assessment framework for emergency medicine specialist examinations.

In South Africa, postgraduate emergency medicine training spans 4 years, culminating in specialist licensing examinations administered by the CMSA. As depicted in Figure 1, the College of Emergency Medicine (CEM) assesses registrars through two multiple-choice question papers, followed by a high-stakes performance exam if required standards are met. This includes four SOE stations, a critical appraisal station and three case-based discussions. This article focuses exclusively on the SOE component, where a single examiner assesses registrars using a criterion-referenced rubric. A different examiner assesses each SOE station.

The CMSA strives to ensure that these assessments align with the competencies required for effective practice. These can be accessed from https://cmsa.co.za/fellowship-of-the-college-of-emergency-medicine-of-south-africa-fcemsa/.

Ensuring that only competent practitioners are certified is a matter of public health and safety, as patients rely on qualified specialists for their care in life-threatening situations. Therefore, improving the fairness, inclusiveness and quality of SOEs contributes directly to patient safety by reducing the risk of certifying underqualified practitioners. While SOEs are crucial for assessing clinical competence, their effectiveness as a sole assessment modality has come under scrutiny, particularly following the shift to web-based formats. While this approach allowed assessments to continue during a time of crisis, it also underscored the limitations of remote assessments, especially in assessing bedside skills such as history taking, physical examination or procedures critical for emergency medicine specialists.^6,7,8^ For instance, when using a web-based SOE, the examiner may struggle to authentically assess a registrar’s ability to interpret non-verbal cues during a patient interaction or perform dynamic procedural tasks such as airway management or central line insertion – skills that are vital in emergency care but difficult to simulate virtually.

A vital issue in SOEs whether in-person or web-based is construct underrepresentation, where the assessment may fail to encompass the range of scenarios needed to assess a candidate’s clinical reasoning and decision-making competence thoroughly.^9^ Emergency medicine’s specialised and demanding nature adds further complexity to SOE design, making it challenging to ensure these assessments reflect the field’s diverse and unpredictable challenges. Thus, a significant challenge is designing SOEs that effectively assess the breadth and depth of clinical reasoning and higher-order decision-making required for competent practice. Additionally, construct-irrelevant variance, where factors unrelated to a candidate’s actual abilities, such as demeanour, language accent or attire, could influence scoring.^10^ Structured oral examinations may be particularly susceptible to these biases because of their subjective nature and reliance on real-time examiner judgement.^3^ This highlights the potential for unconscious bias in the assessment process, which could unfairly advantage or disadvantage certain registrars, undermining the fairness and inclusiveness of the certification process.^11,12,13^ Addressing these concerns is essential to ensure that registrars, irrespective of background, are accurately assessed based on their clinical reasoning, decision-making and problem-solving skills.^12^

In South Africa, the legacy of apartheid continues to influence education and assessment systems.^12^ Decades of segregation have fostered unconscious biases shaped by race, language and socio-political hierarchies, which can affect how registrars are assessed.^12^ These biases, whether related to language accents or perceived cultural differences,^13,14^ may disadvantage registrars from underrepresented backgrounds during assessments like SOEs.^12^ Research in South African medical education has shown that students from historically disadvantaged groups often experience stereotype threat and implicit bias in clinical assessments, which can negatively impact performance and confidence.^12,13^ Efforts by the CMSA to diversify examiner panels and standardise assessment processes reflect a commitment to reducing this type of bias. However, further work is needed to ensure equitable assessment across the diverse candidate pool.

A recent study by Burch et al. reported high acceptability and minimal technical challenges with web-based SOEs for postgraduate certification, primarily from the registrars’ perspective.^5^ However, positive candidate feedback alone does not address whether SOEs adequately assess the complex competencies required in emergency medicine. Notably, 38% of registrars indicated that the exams failed to effectively assess higher-order cognitive abilities, and 33% felt the case scenarios were unsuitable for assessing entry-level specialists.^5^ These findings underscore the importance of improving question design to target higher-order thinking and enhancing examiner training to ensure critical competencies are accurately assessed. Thus, an exploration of the perspectives of both registrars and examiners was considered necessary to provide insights into ways to improve SOEs.

**FIGURE 2:**
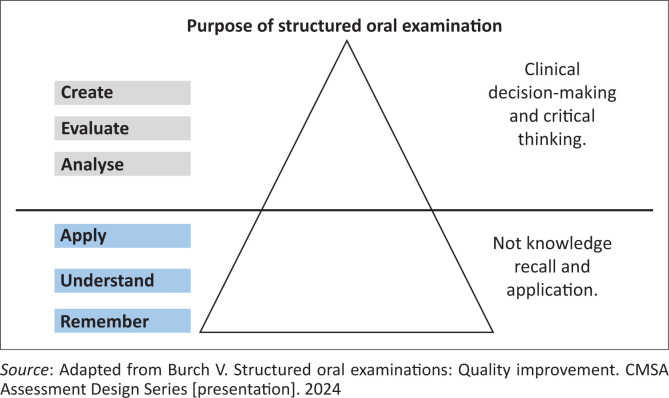
Bloom’s taxonomy of learning.

This study is underpinned by Bloom’s taxonomy,^14^ which emphasises higher-order thinking in learning and assessment. As illustrated in Figure 2,^15^ Bloom’s taxonomy classifies cognitive skills into six levels, with the highest three – evaluate, analyse and create – being most relevant to SOEs. These skills extend beyond factual recall, requiring critical assessment, interpretation and application of knowledge in complex clinical scenarios.^14^

In SOEs, registrars should^15^:

- **Evaluate:** Make informed clinical judgements, weighing diagnostic and therapeutic options.
- **Analyse:** Justify decisions by critically interpreting clinical data.
- **Create:** Formulate action plans that reflect clinical reasoning, such as recommending treatment or further investigations.

Integrating Bloom’s taxonomy into SOEs reinforces the role of the assessment in assessing not just what registrars know but how they use that knowledge to make sound clinical decisions and deliver patient-centred care.^15^

**FIGURE 3:**
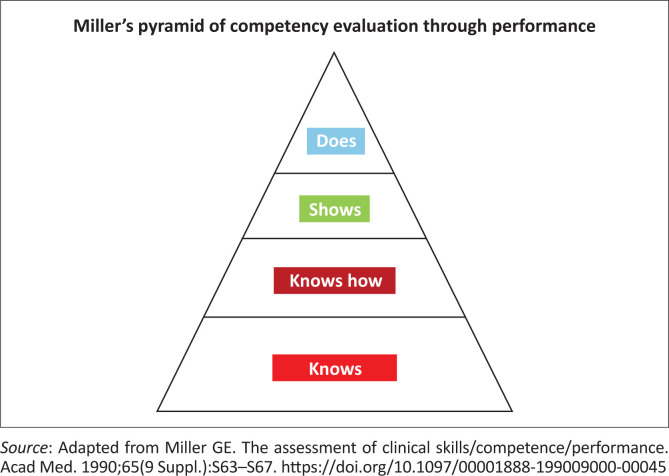
Miller’s pyramid of clinical competence.

This study is also guided by Miller’s pyramid^16^ a recognised framework for assessing clinical competence through four progressive levels: foundational knowledge (‘knows’), competence (‘knows how’), skill demonstration (‘shows how’) and real-world application (‘does’), as illustrated in Figure 3. While SOEs effectively assess the lower levels, they fail to assess real-world clinical performance, which is best measured at the ‘does’ level through direct observation and workplace-based assessments (WBAs). Thus, integrating WBAs with SOEs provides a more comprehensive assessment of a candidate’s readiness for independent practice.

Incorporating both Miller’s pyramid and Bloom’s taxonomy provides a comprehensive framework for assessing different dimensions of learning and competence in SOEs. While Miller’s pyramid emphasises the transition from knowledge to practical application,^16^ Bloom’s taxonomy complements it by focusing on the cognitive complexity required to make sound clinical decisions.^14^

The literature has coalesced around core principles that form the foundation of effective assessment practices.^16^ These guiding principles encompass a range of factors, including validity, reliability, fairness, feasibility, educational impact, authenticity and feedback.^16^ Ultimately, to influence diverse dimensions of learning for as many students as possible, adopting multiple dimensions of assessment, namely assessment systems, rather than adjusting individual assessment events is recommended.^16^

This study sought to address the gap in understanding SOEs within the Fellowship of the College of Emergency Medicine (FCEM) examination conducted by the CMSA. Despite feedback raised by both students and examiners regarding assessment practices in emergency medicine between 2021 and 2023,^8^ including issues of ‘failure to fail’,^14^ inconsistent examination content and the quality of questions, research delving into the perceptions of registrars and examiners within the specialised field of emergency medicine remains notably lacking.

The central *research question* guiding this study was: What are the perceptions and experiences of registrars and examiners in the field of emergency medicine regarding SOEs?

The *aim* of this study was to explore and understand the perceptions and experiences of registrars and examiners in the field of emergency medicine regarding SOEs.

The *objectives* of the study were to:

- explore the perspectives of registrars and examiners regarding SOEs as an assessment practice.
- explore insights from registrars and examiners to gain a deeper understanding of how SOEs could be enhanced in the context of emergency medicine.

The findings of this research have the potential to impact emergency medicine education, both nationally and internationally, by informing the development of more effective assessment practices that accurately reflect the competencies required for emergency medicine practice. In addition, by contributing to broader discussions about assessment in high-stakes medical specialities, this study aimed to influence curriculum design and assessment policy, ultimately leading to the preparation of better-equipped emergency medicine specialists.

## Research methods

### Research design

This study employed a qualitative exploratory design.^17^ This design was chosen to gain in-depth insights into participants’ nuanced experiences and perspectives, which are not easily captured through quantitative methods.^17,18^ In addition, the study is grounded in an interpretivist paradigm, which is ideally positioned to capture the subjective experiences of those directly involved – registrars and examiners. The interpretivist approach acknowledges that reality is socially constructed and that social realities are co-constructed by individuals, making it essential to understand participants’ experiences within their specific contexts.^18,19^

### Population and sampling

The study targeted a group of registrars and examiners involved in a single recent FCEM II examination. A total of 13 participants, including six registrars and seven examiners, agreed to participate. This sample was drawn from a larger pool of 22 registrars and 20 examiners, providing a meaningful cross-section of individuals with direct experience in the examination process. Data collection was conducted approximately 6 months after the examination sitting to ensure experiences were still vivid while allowing for some reflection.

### Recruitment strategy

A combination of convenience and voluntary response sampling was used to recruit participants. Convenience sampling involves selecting participants based on availability and ease of access, while voluntary response sampling relies on participants self-selecting to participate.^17,18^ Recruitment was facilitated through the CMSA. After receiving permission from the CMSA and ethics approval from Stellenbosch University S24/02/029, invitations (which included the informed consent form and information related to the study) were emailed to examiners and registrars by the CMSA. All participants who responded were included in the study. They voluntarily contacted the research team to express their interest.

### Sampling strategy

In determining the sample size, the concept of ‘information power’ proposed by Malterud, Siersma and Guassora^20^ was adopted as an alternative to the conventional ‘data saturation’ concept.^21^ Unlike the notion of data saturation, which is often associated with a positivist approach and implies exhaustive comprehension, ‘information power’ emphasises the relevance and richness of the data collected rather than the sheer quantity.^20,21^ The use of the ‘information power’ framework ensured that the sample size was appropriate for addressing the research questions and aligned with the study’s interpretivist methodological orientation.^20,21^ Unlike data saturation, which might imply a final limit or exhaustive coverage, ‘information power’ focuses on the data’s depth, relevance and specificity, which are critical for achieving conceptual depth and theoretical sufficiency.^20,21^ The sample was considered sufficient because the research question was focused, the interviews produced rich and detailed responses, and the data were analysed in depth across participants. These factors ensured that the study could generate meaningful insights without needing a larger number of participants.

### Data generation

Semi-structured interviews were used to generate data, providing a confidential setting that allowed participants to share their experiences without the influence of others, which can sometimes occur in group settings. The interview questions (Appendix 1) were adapted from Boulais et al.,^4^ with adjustments made to align with the context of emergency medicine SOEs. Piloting the interview guide with a registrar and an examiner allowed for reflection on and refinement of the questions through discussions between the interviewer, researcher and supervisor. For example, an initial broad question, ‘Tell us about your experiences with SOEs in emergency medicine’, was revised to ‘Does your experience with SOEs evoke any memories, whether positive or negative?’ This change encouraged participants to delve into the emotional aspects of SOEs, leading to more reflective responses.

Interviews were conducted by a trained research assistant to ensure neutrality. The interviews were conducted online via Microsoft Teams, lasting 30–45 min each, with audio recordings made for transcription. Online interviews offered logistical benefits but also posed challenges in observing non-verbal cues. To mitigate this, the interviewer used active listening techniques to capture tone and pauses. Follow-up questions were employed to explore underlying emotions and clarify responses. Anonymity and data security were prioritised by assigning alphanumeric codes to transcriptions and storing the data in password-protected cloud storage, with access limited to the research team.

### Data analysis

The audio recordings were transcribed using Microsoft Teams’ transcription feature, with the transcripts carefully reviewed for accuracy and then verified by the participants. Data analysis followed *Braun and Clarke’s six-phase reflexive thematic analysis (RTA)*,^22,23^ a method well suited to the study’s interpretivist paradigm. This approach allowed for the development of themes across cases after coding the entire data set, ensuring that the themes accurately reflected the participants’ nuanced experiences. While the analysis was predominantly inductive, some deductive elements were applied to ensure that the themes were aligned with the research question and provided meaningful insights.

Thematic analysis was conducted over six stages. Initially, the researcher was immersed in the data by repeatedly reading the transcripts and cross-checking them against the audio recordings for accuracy. Initial codes were generated to identify patterns and features of interest within the data – for example, repeated mentions of stress or anxiety were initially coded as ‘emotional strain’. These codes were continually refined and organised into broader categories. Codes such as ‘exam nerves’, ‘pressure from familiarity’ and ‘fear of failure’ were then grouped into a category labelled ‘emotional intensity of SOEs’. The categories were grouped into overarching themes using visual displays and colour coding. The themes were reviewed and adjusted by the research team throughout the process to ensure they were well supported by the data. Each theme was revisited and refined by reviewing the supporting data extracts to ensure coherence within themes and clear distinction between them. Disagreements in coding or theme development were resolved through reflective discussions with the supervisor, leading to collaborative refinement and consensus on final themes. Finally, a comprehensive report was constructed, connecting the themes to the research questions and providing a detailed narrative of the participants’ experiences.

### Trustworthiness

To ensure *trustworthiness*, credibility, transferability, dependability and confirmability were rigorously applied, as suggested by Guba and Lincoln.^19^ Credibility was enhanced through participant verification and discussions with the co-author, who reviewed the coding process. Transferability was ensured by providing rich descriptions of the study’s context and participant experiences. Dependability was achieved by consistently applying the research methodology, with all processes thoroughly documented. Confirmability was maintained through an audit trail and reflexive journaling to minimise personal biases. Although data triangulation was not employed, analytic triangulation was used to enhance the credibility of the analysis.^21^ This involved the supervisor independently reviewing the coding and theme development to minimise individual researcher bias and support interpretive rigour. To manage reflexivity more deliberately, the researcher maintained a reflexive journal throughout data collection and analysis, noting assumptions, emotional responses and positionality. These reflections were discussed in regular debriefing sessions with the supervisor to ensure critical engagement with emerging interpretations and reduce the risk of bias influencing the findings. Additionally, inter-coder reliability was strengthened through co-author cross-checking, and member checking was utilised to ensure the findings accurately represented the participants’ perspectives. Interview transcripts were emailed to participants for verification.

### Ethical considerations

The study complied with ethical guidelines, adhering to the principles of beneficence, justice and respect for persons.^24^ Ethical approval was obtained from the Human Research Ethics Committee of the Faculty of Health Sciences at Stellenbosch University (No. S24/02/029). Additionally, permission was obtained from the CMSA NPC to conduct the study. The principle of justice was upheld by ensuring equitable access to participation, with fair recruitment practices facilitated through the CMSA, ensuring that all eligible participants had an equal opportunity to be included in the study.

Recruitment was conducted in full compliance with the Protection of Personal Information Act (POPIA), ensuring that participants’ privacy and data protection were upheld throughout the study. Invitations to participate were sent via email from the CMSA, and the first 6–10 respondents from each group who expressed interest were included in the study. Participants were informed of the voluntary nature of their participation, with assurances that their decision to participate would not affect their professional standing or relationships within the college, thereby upholding the principle of respect for persons.

Informed consent was obtained from all participants after providing detailed information about the study’s purpose, procedures and their rights, including the choice to withdraw at any time without consequences. The principle of beneficence was ensured by minimising risks to participants, respecting their time and comfort and fostering a safe and supportive environment during the interviews. Confidentiality and anonymity were maintained by anonymising data, using pseudonyms and securely storing all recordings and transcriptions on a password-protected system, with access limited to the research team. Data will be stored for 5 years for potential future verification before being permanently deleted. Participants were offered a food voucher worth R200 each to show appreciation for their time. This gesture was carefully balanced with the ethical considerations of voluntary participation and non-coercion.

## Results

Following the reflective thematic analysis of the data from both registrars and examiners, four main themes were developed (Table 1):

- Theme 1: The emotional battleground – Navigating anxiety in SOEs
- Theme 2: Fairness under the microscope –The relevance of SOE content
- Theme 3: More than just exams – The push for continuous assessment
- Theme 4: Behind the scenes – The importance of examiner training

**TABLE 1:** Themes arising from the study.

Theme	Subtheme
1. Emotional battleground: Navigating anxiety in SOEs	1.1. Walking the tightrope of stress 1.2. The familiarity dilemma: Comfort or pressure?
2. Fairness under the microscope: The relevance of SOE content	2.1. The theory: Practice tug of war 2.2. Cultural and language conundrum
3. More than just exams: The push for continuous assessment	3.1. Moving beyond the one-time show
4. Behind the scenes: The importance of examiner training	4.1. Bridging the training gap 4.2. Digital divide: Training for virtual SOEs

SOE, structured oral examinations.

These themes offer insights into the key challenges and opportunities for improving SOEs as a high-stakes assessment method. The themes are interwoven, particularly in relation to improving assessment methods that reduce emotional stress, ensure fairness and align more closely with critical reasoning, higher-order thinking and the practical requirements of emergency medicine. Each theme is introduced with relevant sub-themes, supported by direct participant quotes.

### Thematic analysis: Unpacking the high-stakes world of structured oral examinations

#### Theme 1: The emotional battleground – Navigating anxiety in structured oral examinations

Structured oral examinations place a significant emotional burden on registrars, often resulting in heightened anxiety and stress. This theme explores the emotional challenges registrars face, particularly under the pressure of high-stakes assessments. Two key sub-themes were developed: Sub-theme 1.1: *Walking the tightrope of stress*, which addresses the overwhelming pressure registrars experience during the exam process, and sub-theme 1.2: *The Familiarity dilemma – Comfort or pressure?* These sub-themes explores the dual role of examiner familiarity in either alleviating or amplifying stress depending on the candidate’s perspective.

**Sub-theme 1.1: Walking the tightrope of stress:** The emotional weight of SOEs was palpable, with registrars describing the immense pressure of high-stakes exams. This stress stems from the fear that the exam determines their future career trajectory, leaving them constantly on edge. One candidate explained:

‘The whole process is incredibly stressful. You feel like everything you’ve worked for is on the line, and that can be overwhelming.’ (Reg 1)

The structured nature of SOEs, intended to standardise the process, paradoxically contributes to the pressure by requiring registrars to answer a set number of questions within a limited time. The time constraints during these exams were described as particularly stressful, adding an extra layer of difficulty, as one candidate noted:

‘When you only have ten minutes, it adds to the stress.’ (Reg 2)

Many registrars found that preparation, particularly through mock exams, was critical in mitigating this anxiety. By simulating the exam environment, they were better able to manage time pressure and anticipate the structure of the exam. As one registrar noted:

‘Mock exams really helped. It gave me a sense of what to expect and made me feel more prepared on exam day.’ (Reg 4)

**Sub-theme 1.2: The familiarity dilemma – Comfort or pressure?:** Familiarity with examiners during the SOE had varied effects on registrars. For some, seeing familiar faces provided reassurance, making the exam environment less intimidating. As one registrar described:

‘Familiarity made the environment less daunting and gave me a sense of calm, allowing me to focus on the task at hand.’ (Reg 6)

However, for others, it had the opposite effect, heightening the pressure to perform and meet the expectations of people they knew. One registrar expressed this tension, noting that:

‘… seeing familiar faces made me more anxious because I didn’t want to disappoint them.’ (Reg 1)

This contrast highlights how familiarity can either alleviate or amplify the emotional burden of SOEs.

Examiners, too, faced challenges in maintaining objectivity when assessing registrars with whom they were familiar. The potential for leniency or bias in these situations was a recognised concern. One examiner acknowledged that they took active measures to remain reflexive throughout the process and:

‘… knowing the candidate personally can make it difficult to maintain complete objectivity.’ (Examiner 6)

This reflects the awareness among examiners of the need to consciously mitigate bias to ensure fairness in their evaluations.

#### Theme 2: Fairness under the microscope – The relevance of structured oral examinations content

This theme explores how participants understood fairness in relation to the content of the SOE, not only its administration. Rather than focusing solely on procedural fairness (e.g. consistency in exam delivery), participants highlighted concerns about whether the questions themselves were relevant, meaningful and representative of real-world emergency medicine. In this sense, fairness was tied to the substantive quality of the questions – whether they allowed registrars to demonstrate the clinical reasoning and decision-making skills that are central to specialist practice. Two key sub-themes were developed: Sub-theme 2.1: *The theory-practice tug of war*, which explores tensions between theoretical questioning and practical clinical reasoning, and sub-theme 2.2: *The cultural and language conundrum*, which highlights how language and phrasing can influence candidates’ ability to engage with the material.

**Sub-theme 2.1: The theory-practice tug of war:** Registrars had differing views on the fairness of SOE content. Some valued the way the questions challenged their critical thinking, particularly in relation to emergency medicine. As one registrar noted:

‘Some of the questions made me think through real-life scenarios, forcing me to apply my knowledge in a way that reflects what happens in emergency medicine.’ (Reg 4)

However, others felt that certain questions were too focused on theoretical knowledge and disconnected from the practical realities of day-to-day clinical work. One registrar remarked:

‘Questions seemed more about testing theoretical knowledge than reasoning.’ (Reg 5)

This division underscores the varying perceptions of how well SOE content aligns with clinical practice.

Examiners echoed this concern, emphasising the need to design questions that assess knowledge, critical reasoning and higher-order thinking. One examiner remarked:

‘The questions should reflect what we do in the emergency department; otherwise, they miss the point.’ (Examiner 2)

Underscoring the importance of aligning exam content with the practical demands of emergency medicine, another examiner acknowledged:

‘… in some cases, we’re not testing critical thinking or higher-order reasoning enough.’ (Examiner 5)

**Sub-theme 2.2: The cultural and language conundrum:** Non-native English speakers sometimes struggled with the phrasing of questions, adding an additional layer of difficulty. This language barrier was acknowledged by both registrars and examiners, with some registrars expressing that it affected their ability to demonstrate their knowledge fully. One registrar explained that:

‘… the wording of some questions was really tough, and it made it hard to show what I actually knew.’ (Reg 2)

Examiners also recognised the impact, as one remarked:

‘Sometimes the phrasing of questions can be challenging for non-native (English) speakers, and that impacts performance.’ (Examiner 1)

This highlights the need for better training in question design to ensure that language does not unfairly disadvantage registrars.

#### Theme 3: More than just exams – The push for continuous assessment

This theme explores the growing advocacy for integrating continuous assessment methods into the evaluation process, mainly through WBAs. Sub-theme 3.1: *Moving beyond the one-time show* was dominant throughout the data, highlighting the desire for a more comprehensive and ongoing assessment of clinical competence and moving away from the high-stakes nature of a single exam. Both registrars and examiners emphasised the value of WBAs in providing a more accurate reflection of real-world practice in emergency medicine.

**Sub-theme 3.1: Moving beyond the one-time show:** There seemed to be a desire for continuous assessment through WBAs. Registrars argued that WBAs would offer a more comprehensive assessment of their abilities in real-world settings, alleviating the pressure of a single high-stakes exam. Workplace-based assessments were viewed as a more accurate reflection of the daily responsibilities and skills required in emergency medicine. One registrar explained:

‘Continuous assessment would be a better way to gauge our readiness, rather than just one high-stakes exam at the end.’ (Reg 2)

Examiners echoed this sentiment, emphasising that combining SOEs with WBAs would provide a more holistic picture of a candidate’s readiness for independent practice. As one examiner noted:

‘… the exam should carry less weight with more focus on work[*place*]-based assessments.’ (Examiner 2)

This approach was seen as better balancing the assessment of theoretical knowledge and practical application in emergency medicine. Another examiner added:

‘Longitudinal assessments like WBAs are definitely a stronger and more important approach to assessment.’ (Examiner 3)

#### Theme 4: Behind the scenes – The importance of examiner training

This theme addresses the critical need for enhanced examiner training to foster a culture of fair and rigorous assessment. Two sub-themes emerge: Sub-theme 4.1: *Bridging the training gap*, which highlights the necessity of structured training to craft better questions that test higher-order thinking, manage examiner familiarity with registrars and mitigate unconscious bias and Sub-theme 4.2: *The digital divide – Training for virtual SOEs*, focusing on the challenges posed by the shift to online assessments and the need for training in virtual engagement techniques. Together, these efforts aim to build a culture of assessment that upholds fairness.

**Sub-theme 4.1: Bridging the training gap:** The need for improved examiner training was a recurring concern. Many examiners acknowledged that their current training was insufficient to ensure fair and effective assessments, particularly in crafting questions that challenge critical thinking and reasoning. One examiner admitted:

‘The existing process doesn’t provide enough preparation and observing one SOE before becoming an examiner is definitely not enough.’ (Examiner 2)

There were widespread calls for more structured and ongoing professional development, with a focus on ensuring that SOE questions adequately tested higher-order cognitive skills:

‘… we need a more structured approach to training with ongoing professional development and clear guidelines on what makes a good SOE question.’ (Examiner 5)

Training was also seen as essential in helping examiners navigate the complexities of assessment, as unconscious bias could influence their judgements. One examiner noted:

‘There’s always going to be bias – what a candidate looks like or what they sound like … umm, there’s always going to be that subliminal bias.’ (Examiner 4)

Structured training was emphasised to mitigate these biases:

‘… yeah, we need a more structured approach to training, not only to ensure fair assessments but also to prevent unconscious bias in the process.’ (Examiner 2)

**Sub-theme 4.2: The digital divide – Training for virtual structured oral examinations:** The shift to virtual SOEs because of the pandemic introduced a new training need: engaging effectively with registrars in an online environment. Examiners noted that the absence of non-verbal cues made it more challenging to accurately assess registrars and establish rapport. One examiner shared:

‘It’s a lot more difficult to get an impression because you’re just seeing a face on a screen as opposed to the whole person.’ (Examiner 2)

Training in virtual engagement techniques, such as maintaining eye contact through the screen, offering verbal reassurance and managing technical challenges, was recommended to improve the quality of online assessments:

‘… training in virtual engagement techniques, like maintaining eye contact, could make a big difference.’ (Examiner 4)

## Discussion

The specific research question for this study was: What are the perceptions and experiences of registrars and examiners regarding SOEs in emergency medicine? This study sheds light on the intricate dynamics of SOEs within emergency medicine, revealing how emotional, cognitive and contextual factors shape both registrars’ and examiners’ experiences. Principally, the themes speak to the importance of ensuring that SOEs assess clinical reasoning and address the pressures and biases inherent in the assessment process. The findings also reinforce how current SOE practices fall short of assessing the higher-order thinking outlined in Bloom’s taxonomy and the real-world performance represented by the ‘does’ level of Miller’s pyramid. Thus, there are several areas for improvement, including the need for SOEs to be better designed to assess higher-order thinking, the potential benefits of integrating WBA into the FCEM assessment framework and the significance of continuous examiner training to enhance fairness and reliability.

### Emotional and cognitive challenges in structured oral examinations

The emotional burden of SOEs was a dominant theme, with registrars describing the experience as an ‘emotional battleground’ (Registrar 5) because of the high stakes involved. This finding is consistent with prior research on SOEs,^3,4,13^ which highlights the stress associated with oral exams. The structured and time-pressured nature of SOEs, although intended to standardise the process, often exacerbates stress, impairing performance and potentially undermining the validity of the assessment. The reliance on rapid responses rather than thoughtful, well-considered clinical reasoning may lead to superficial evaluations of a candidate’s competence.^13,25^ In addition, the emotional burden of SOEs may impair performance, particularly under strict time constraints and high-stakes pressure.^25^ The stress of high-stakes testing may also hinder the assessment of critical reasoning,^25^ pointing to the need for improvements in SOE design.

### Addressing gaps in structured oral examinations design: From theoretical knowledge to practical competencies

A critical concern raised by some registrars in this study was the disconnect between SOE scenarios and the realities of clinical practice. Many registrars expressed frustration that the examination questions often emphasised theoretical knowledge rather than practical problem-solving skills, which are crucial in emergency medicine.^3^ This issue aligns with Bloom’s taxonomy,^14^ which stresses the need to assess higher-order cognitive abilities such as analysis, evaluation and creation. In emergency medicine, clinicians must be able to apply their knowledge dynamically, under pressure and in complex situations.

A key issue identified in this study, and one that is echoed in the broader literature, is construct underrepresentation.^9^ This occurs when SOEs fail to adequately assess the breadth of clinical reasoning and decision-making competencies required in emergency medicine. As noted by Boulais et al.^4^ and Chudnofsky et al.,^3^ SOEs, because of their structured and time-limited nature, may not fully reflect the unpredictable and fast-paced environment of emergency medicine, where clinicians must adapt to evolving clinical scenarios. This limitation points to the need for a more robust SOE design that better mirrors the complexities of real-world practice, addressing validity and reliability concerns. It is thus crucial to revise SOE content to better reflect the practical realities of clinical practice. Training emergency medicine examiners in developing and reviewing exam scenarios will ensure they are realistic and representative of the field’s challenges. Rather than focusing solely on lower-order cognitive skills such as recall and comprehension, the content should shift towards higher-order thinking as outlined by Bloom’s taxonomy, including application, analysis, synthesis and evaluation. The emphasis should be on critical decision-making and thinking to assess registrars’ competencies thoroughly. Additionally, questions should target areas requiring higher-order cognitive skills, such as analysing complex clinical scenarios, evaluating treatment options and applying theoretical knowledge in practical settings. This ensures the assessment identifies knowledge gaps that could compromise patient care, moving beyond simple recall to assess registrars’ readiness for real-world challenges.

This finding reinforces and extends previous studies that have questioned the alignment of SOE content with clinical realities. While studies such as Boulais et al.^4^ and Chudnofsky et al.^3^ have identified theoretical overemphasis, this study adds nuance by showing how this misalignment is experienced by both registrars and examiners in a resource-variable South African context. The challenges of limited exposure, diverse patient presentations and local clinical constraints further emphasise the need for content that reflects real-world emergency medicine practice.

This disconnect also relates to the phenomenon of ‘failure to fail’,^26^ where registrars may pass based on their ability to recall theoretical knowledge but fail to demonstrate the higher-order thinking and clinical reasoning necessary for safe, independent practice. The findings reinforce concerns introduced in the literature, such as those by Daly et al.,^26^ that current SOE designs in emergency medicine may inadequately assess these crucial competencies, thereby compromising the validity of the exam. While this concern did not arise directly in the interviews, its absence may reflect limited awareness among examiners of how flawed question design can impact pass or fail decisions. Many examiners are experienced clinicians but may lack formal training in assessment principles, which could explain why the consequences of poorly constructed SOEs were not explicitly acknowledged. This further supports the need for structured examiner development and more deliberate focus on assessment validity.

Additionally, the issue of construct-irrelevant variance,^10^ such as a candidate’s demeanour, language proficiency or other factors unrelated to their clinical abilities, was a significant concern. As noted by Niehaus et al.,^13^ SOEs are particularly susceptible to bias, given their reliance on real-time examiner judgements. This study highlights the potential for unconscious bias to influence assessments, especially in a multicultural and multilingual context like South Africa, where examiners’ biases related to language and cultural background may unfairly affect candidate performance.^14^ Addressing these biases is critical to ensuring that SOEs remain fair and inclusive.^16^

### The role of workplace-based assessments

In light of these limitations, the integration of WBAs is strongly supported by this study and previous research.^6,7^ Workplace-based assessments offer a more holistic and contextualised assessment of clinical competence by assessing registrars in real-time, authentic environments.^6^ This approach aligns with Miller’s pyramid,^16^ which emphasises that true clinical competence is demonstrated not only through knowledge but also through performance in real-world practice. Workplace-based assessments can assess competencies such as communication, teamwork and professionalism – skills that are often difficult to capture in an SOE setting.

Workplace-based assessments improve the authenticity of assessments by offering a real-world perspective. Combining SOEs with WBAs creates a more balanced assessment framework, capturing the full range of clinical competencies needed in emergency medicine. As supported by Sidhu and Fleming,^27^ this approach ensures that registrars are assessed on their consistency over time, reflecting their day-to-day clinical practice and addressing both educational impact and fairness in the assessment process. This combined approach can reduce the pressure of a single high-stakes exam and offer a more accurate reflection of a candidate’s readiness for independent practice by providing more assessment opportunities (data points) and possibly the opportunity to be assessed by different assessors.^28^

### Continuous examiner training and mitigating bias

A recurring theme in this study was the need for continuous examiner training to address two major concerns: improving question design to assess higher-order thinking and mitigating unconscious bias both in favour of and against the candidate. Firstly, examiner training should focus on developing questions that challenge critical thinking and clinical reasoning rather than merely testing factual recall. Incorporating Bloom’s Taxonomy^14^ into the training process can help ensure that questions are designed to assess complex cognitive processes.

Secondly, training programmes should address unconscious bias, particularly biases related to race, language and cultural background. As highlighted by Mrara et al.^12^ and Niehaus et al.,^13^ biases in examiner judgements can significantly impact the fairness of assessments. Training examiners to recognise and mitigate these biases is crucial for maintaining the integrity of SOEs. This study underscores the importance of structured, ongoing examiner training that equips examiners to design reliable and valid assessments that accurately assess the diverse competencies required in emergency medicine. Training programmes should also include skills for virtual engagement to address the unique challenges of online assessments, ensuring that examiners are fully equipped to assess registrars effectively in digital formats.

### Practical implementation and broader considerations

In the South African context, these findings take on particular urgency. The assessment landscape is shaped by wide disparities in clinical exposure between urban and rural training sites, ongoing efforts to redress historical inequities and a linguistically diverse registrar population.^12^ These factors amplify the need for examiner training that explicitly addresses language bias and cultural responsiveness, as well as for assessment methods like WBAs that can accommodate local variation in caseload and supervision. The study’s insights into fairness, question design and the limits of SOEs are therefore directly relevant to current national conversations around transformation, inclusivity and maintaining rigour in specialist certification. Extrapolating findings to other disciplines will require tailoring to their specific contexts, ensuring alignment with their unique challenges and training goals.

Furthermore, assessment practices are dynamic and continue to evolve, particularly with the rise of WBAs. The pandemic has driven flexible assessment practices, highlighting the need for adaptable approaches that remain relevant in a post-pandemic world. Furthermore, the possibility of future pandemics or similar global disruptions underscores the importance of maintaining flexibility and resilience in assessment strategies to ensure continuity in diverse circumstances. In addition to the CEM, other stakeholders, such as regulatory bodies (Health Professions Council of South Africa, healthcare institutions and universities), must be involved to ensure these recommendations are robust and widely applicable.

To support implementation, Table 2 summarises actionable recommendations based on the study’s findings, grouped into three focus areas: examiner training, SOE question design and integration of WBAs.

**TABLE 2:** Summary of practical recommendations for educational practice in emergency medicine assessment.

Area of focus	Recommendation	Rationale
Examiner training	Develop structured training modules tailored to emergency medicine.	Ensures examiners understand the clinical context and cognitive expectations of SOE stations.
	Include training on unconscious bias and cultural responsiveness.	Promotes fairness and inclusivity, especially in multilingual and diverse South African settings.
	Offer cross-disciplinary workshops to share best practices.	Fosters inter-specialty learning and alignment of quality standards across the CMSA.
	Establish ongoing refresher courses.	Reinforces assessment literacy and supports evolving conceptions of competence.
SOE question design	Align questions with Bloom’s higher-order taxonomy (analyse, evaluate, create).	Encourages deeper clinical reasoning and decision-making, rather than rote recall.
	Include real-life, contextually relevant clinical scenarios.	Improves authenticity and aligns assessments with emergency medicine practice.
	Pre-review all questions for language clarity and cultural inclusivity.	Reduces construct-irrelevant variance, particularly for non-native English speakers.
	Establish question design panels with diverse clinician input.	Promotes relevance and fairness across practice settings and demographics.
Integrating WBAs with SOEs	Use WBAs to assess ‘does’ level of Miller’s pyramid in real-world settings.	Captures authentic, longitudinal performance and complements the snapshot view of SOEs.
	Implement WBAs across all clinical rotations using standardised tools.	Ensures comparable data across training sites with different resources and supervision.
	Involve multiple assessors over time to reduce bias and enhance credibility.	Improves reliability and breadth of performance assessment.
	Combine formative WBAs with high-stakes SOEs to inform progression decisions.	Supports holistic and programmatic assessment, balancing one-time performance with ongoing competence.

SOE, structured oral examinations; CMSA, Colleges of Medicine of South Africa; WBA, workplace-based assessments.

### Future research directions

Several avenues for future research are recommended to develop further and refine assessment practices in medical education:

#### Long-term impact of complementing structured oral examinations and workplace-based assessments in emergency medicine assessment

As assessment methods evolve, understanding how the combination of SOEs and WBAs can provide a more comprehensive assessment of clinical competence will be vital in shaping the future of medical education. Successful implementation of WBAs alongside SOEs will require structured change management, including early engagement of clinical faculty, alignment with institutional and regulatory frameworks and clear articulation of how WBAs contribute to registrar development and progression. Faculty development initiatives must equip supervisors with the skills to observe, document and provide feedback consistently while avoiding tokenistic completion of assessment forms. Importantly, integrating WBAs across diverse training platforms will demand attention to standardisation and fairness, particularly in resource-variable settings. Over time, a well-supported WBA system can cultivate a culture of continuous feedback and reflection, providing a richer picture of registrar competence and readiness for independent practice.

#### Challenges and benefits of web-based structured oral examinations

With the rise of virtual assessments, particularly in response to the flexibility demanded by the coronavirus disease 2019 (COVID-19) pandemic, future studies should examine the challenges and benefits of this format in high-stakes settings like emergency medicine. Research could investigate how the lack of in-person interaction affects the assessment of non-verbal communication skills and overall candidate presence – critical factors in clinical practice.

#### The effectiveness of examiner training programmes deserves further exploration

Research that investigates the most effective methods for training examiners – both in technical assessment skills and in mitigating biases – could contribute significantly to the development of best practices in medical education.

### Researcher reflexivity and role in the study

Throughout this research journey, my personal and professional experiences have significantly shaped my perspective. With 15 years of practice as an emergency medicine physician and a focus on medical education research, I brought a unique lens to the study. As someone who navigates the world with a stutter, I have developed a heightened sensitivity to the nuances of verbal communication, particularly in high-pressure situations such as SOEs. Initially, I harboured doubts about the relevance of SOEs in a competency-focused educational landscape. However, I made a deliberate effort to remain open-minded, allowing my assumptions to be challenged. Over time, my perspective shifted, and I began to appreciate SOEs for their potential to assess critical thinking while also recognising areas for improvement. This evolution reflects the transformative nature of qualitative research, where the researcher’s journey plays a crucial role in shaping the findings.

In addition to my personal journey, I navigated the dual role of President of the CEM and researcher. While my insider status afforded me a deep understanding of the assessment culture and access to established professional relationships, it also presented challenges related to power dynamics and potential biases. To maintain the integrity of the research, I employed several strategies. An independent interviewer was appointed to conduct semi-structured interviews, allowing participants to share their experiences openly, free from the influence of perceived power imbalances. Furthermore, the study took place 6 months after the SOEs, giving participants space to reflect without the immediate impact of examination outcomes.

Reflexivity was central to my approach. I maintained a reflexive journal throughout the study, critically documenting my thoughts and assumptions. Aware that my roles as President, examiner and convenor could shape my understanding, I engaged in regular debriefing sessions with my supervisor, critically reflecting on how my insider perspective might influence the analysis – particularly regarding themes such as assessment fairness. My supervisor also independently verified the data coding to ensure that interpretations were grounded in the data, not my preconceptions. Additionally, I sought diverse perspectives from participants and incorporated member checking, where both examiners and registrars reviewed transcripts to confirm that their experiences were accurately represented.

These reflexive and methodological strategies were critical to ensuring the rigour of the study, mitigating potential biases and providing an authentic understanding of the assessment practices within the CEM. By continuously reflecting on my role and securing independent validation, I was able to safeguard the integrity and authenticity of the research findings.

## Limitations

Reflecting on our research design and guided by Lingard’s approach to considering research limitations, we recognise several factors that may influence the interpretation and application of our findings.^27^ Our study focused on a single college and a specific group of registrars and examiners within emergency medicine, providing a rich, context-specific understanding of SOEs. While this focus allowed for deep insights, it may also limit the transferability of our findings to other medical specialities or educational contexts. By offering thick descriptions and employing relevant theoretical frameworks, we aim to assist readers in assessing the potential transferability of the principles of this study to their own settings.

The timing of our study, conducted 6 months after the SOEs, introduces potential recall bias. Participants had time to reflect on their experiences, which may have enriched the data but could also differ from their immediate post-examination perceptions. Future research could compare immediate and delayed reflections to explore how perceptions of SOEs evolve. In addition, voluntary response sampling relies on participants self-selecting to participate, which can introduce bias, as those with a particular interest in the topic may be more likely to respond. Finally, the voluntary nature of participation could have introduced self-selection bias, with participants holding strong opinions, which could potentially skew findings.

Additionally, using an independent interviewer was intended to reduce potential bias arising from the researcher’s position as President of the CEM. While this approach likely enhanced objectivity, it may have limited the depth of insider knowledge during the interviews.

## Conclusion

In conclusion, this study provides valuable insights into the perspectives of registrars and examiners regarding SOEs in emergency medicine, highlighting the need to revise SOE content to assess higher-order thinking and clinical reasoning better. Integrating WBAs emerged as a key complementary approach, offering a more comprehensive evaluation of registrars by capturing real-world performance. Continuous examiner training is essential to improve question design and mitigate unconscious biases that may impact the fairness of assessments. Together, these findings underscore the importance of developing assessment systems that are not only valid and reliable but also equitable and reflective of the practical demands of emergency medicine, ultimately contributing to the preparation of competent specialists and safeguarding patient care.

## Appendix 1

### Interview prompts for participants

#### For registrars

1. Tell us about your experiences with structured oral examinations (SOEs) in emergency medicine.
  Can you give an example?
  - Probing: Why do you feel this way?
  - (prompt)? What were the barriers/constraints? Why do you see this as a barrier? How did this make you feel?
  - Adjusted: around emotions associated with SOE.
2. How well do the exam questions match what you do in emergency medicine? If they don’t, what could make them better?
  - Probing: Can you share specific examples of questions that didn’t match and explain how they could be improved?
3. How did you experience the interaction with your examiner during your exams’? Why is this interaction important?
  - Probing: Can you describe a situation where this interaction was particularly helpful or any suggestions for making it more effective?
4. What suggestions can you make to improve future assessments? Any suggestions?
  - Probing: Can you provide specific recommendations based on your experiences and insights?
5. Are there things about exams that you think we should keep because they make assessments better?
  - Probing: Could you explain why these aspects should be retained for the benefit of future assessments?

#### For examiners

1. What do you think about SOEs in emergency medicine?
  - Probing: Can you share specific observations that influenced your opinion on the quality of exams?
2. What challenges have you faced as an examiner in exams? How did you handle them?
  - Probing: How have these challenges affected your role as an examiner, and can you elaborate on the strategies you found effective?
3. Are the exam questions aligned to the knowledge, skills and attitudes expected in emergency medicine? Can you give examples of good or not-so-good questions?
  - Probing: Could you provide specific examples to illustrate your point and suggest ways to improve question quality?
4. What recommendations can you make to improve the assessment experience/outcome/structure?
  - Probing: What specific improvements do you have in mind, and how do you believe they would enhance the questions?
5. Can you suggest any new ideas or methods to improve the fairness and quality of the questions?
